# Enhanced Electrical Performance of Monolayer MoS_2_ with Rare Earth Element Sm Doping

**DOI:** 10.3390/nano11030769

**Published:** 2021-03-18

**Authors:** Shijie Li, Shidai Tian, Yuan Yao, Meng He, Li Chen, Yan Zhang, Junyi Zhai

**Affiliations:** 1 Center on Nanoenergy Research, School of Physical Science and Technology, Guangxi University, Nanning 530004, China; lishijie@binn.cas.cn (S.L.); tianshidai@binn.cas.cn (S.T.); yaoyuan@binn.cas.cn (Y.Y.); hemeng@binn.cas.cn (M.H.); 2 CAS Center for Excellence in Nanoscience, Beijing Key Laboratory of Micro-Nano Energy and Sensor, Beijing Institute of Nanoenergy and Nanosystems, Chinese Academy of Sciences, Beijing 100083, China; 3 School of Physics, University of Electronic Science and Technology of China, Chengdu 610054, China; 4 College of Nanoscience and Technology, University of Chinese Academy of Science, Beijing 100049, China

**Keywords:** monolayer MoS_2_, CVD growth, Sm doping, electrical performance, FET

## Abstract

Rare earth (RE) element-doped two-dimensional (2D) transition metal dichalcogenides (TMDCs) with applications in luminescence and magnetics have received considerable attention in recent years. To date, the effect of RE element doping on the electronic properties of monolayer 2D-TMDCs remains unanswered due to challenges including the difficulty of achieving valid monolayer doping and introducing RE elements with distinct valence and atomic configurations. Herein, we report a unique strategy to grow the Sm-doped monolayer MoS_2_ film by using an atmospheric pressure chemical vapor deposition method with the substrate face down on top of the growth source. A stable monolayer triangular Sm-doped MoS_2_ was achieved. The threshold voltage of an Sm-doped MoS_2_-based field effect transistor (FET) moved from −12 to 0 V due to the p-type character impurity state introduced by Sm ions in monolayer MoS_2_. Additionally, the electrical performance of the monolayer MoS_2_-based FET was improved by RE element Sm doping, including a 500% increase of the on/off current ratio and a 40% increase of the FET’s mobility. The electronic property enhancement resulted from Sm doping MoS_2_, which led internal lattice strain and changes in Fermi energy levels. These findings provide a general approach to synthesize RE element-doped monolayer 2D-TMDCs and to enrich their applications in electrical devices.

## 1. Introduction

In recent years, there has been an increasing interest in two-dimensional (2D) transition metal dichalcogenides (TMDCs) due to their unique properties and great potential for electronic and optoelectronic applications [1,2]. 2D-TMDCs are a kind of low-dimensional materials that have the formula of MX_2_, where M stands for the transition-metals like Mo, W, and Ti, and X represents S, Se, and Te [3]. Nevertheless, the characteristics presented by 2D-TMDCs are extremely monotonous and limited [4]. Realizing the full potential of 2D-TMDCs in high-performance thin film transistors and to endow some new distinguishing features requires some doping strategies to effectively control their carrier type and to modulate the band gap [5]. Common doping strategies for 2D-TMDCs include substitution doping during growth, ion implantation, and surface charge transfer [6]. However, in these previous doping schemes, ion injection and surface charge transfer in monolayer 2D-TMDC doping were often not stable enough, thus limiting their application [6,7]. Substitution the doping of 2D-TMDCs has been widely explored for materials applications in electronic and optoelectronic [7], as well as room-temperature ferromagnetism, applications [8,9,10]. Transition elements have been used as cationic substitutes for doped 2D-TMDCs, e.g., the Nb ion-doped 2D-TMDCs achieving p-type [7] transport characteristics and the Re ion-doped 2D-TMDCs achieving nearly degenerate n-type doping [11]. Moreover, in recent studies, Fu’s group confirmed ferromagnetism in monolayer MoS_2_ via in situ Fe-doping at room temperature [10] and Pham’s group enhanced tunable ferromagnetism in V-doped WSe_2_ monolayers at 0.5–5 at% V concentrations [9]. Additionally, other transition metal elements in the in situ substitution of MoS_2_-doped for electronic application studies have been demonstrated, such as for Mn [12]. The above research had demonstrated that the doping of transition metal elements is able to tune the electrical, optical, and magnetic properties of 2D-TMDCs [13]. Thus far, transition metal elements have been widely demonstrated in in situ substitution-doped monolayer 2D-TMDCs. However, the doping engineering of atomically thin TMDCs by introducing elements with different atomic valences and atomic configurations, such as RE elements, is still challenging. Currently, a range of difficult issues still exists for in situ RE element substitution-doped large monolayer 2D-TMDCs.

RE elements, which usually exist as trivalent cations, are composed of 15 lanthanides (from lanthanum to lutetium), plus scandium and yttrium [14]. In previous studies, it could be noted that RE ions were commonly doped in traditional insulator or semiconductors [15]. RE elements can also be used as efficient dopants in TMDC materials. Lanthanide (Ln) ions have a rich *f*-orbit configuration that allows them to absorb and emit photons from the ultraviolet to infrared region via the 4*f*-4*f* or 4*f*-5*d* transition, making them candidates for extended 2D-TMDC semiconductor luminescence [15,16]. In addition, RE dopants with unfilled 4*f* energy states and charge-transfer state structures may provide strong spin-orbit coupling to tune the semiconductor properties of the 2D-TMDC’s host material [17]. Furthermore, first principle calculations confirmed the possibility of doping 2D-TMDCs with rare earth elements [18,19]. Currently, progress is being made in the study of RE element-doped 2D-TMDCs films for optical, electronic, and magnetic applications [20]. For example, Qi Zhao et al. made a breakthrough to synthesize MoS_2_:Dy sheets with robust and adjustable ferromagnetic properties at room temperature by a gas–liquid chemical deposition method [17]. Gongxun Bai et al. synthesized a novel 2D system of an Er-doped multilayer MoS_2_ to study NIR-to-NIR down-and up-conversion photoluminescence [16]. However, these studies were based on some thicker-layer 2D-TMDC materials. Later on, Yongxin Lyu et al. fabricated Er-embedded MoS_2_ triangle islands along the in-plane size of up to around 10 μm, which apparently formed single crystals [21]. Additionally, Fu et al. used a salt-assisted sustained-release chemical vapor deposition (CVD) method to grow Eu ion-doped MoS_2_ [22]. Their methods yielded smaller sized samples and tended to introduce new dopant impurities. In addition, Eu and Er element-doped 2D-TMDC research has focused on photoluminescence and ferromagnetic properties, with little reported on the electrical properties of other rare earth element-doped monolayer 2D-TMDCs. Therefore, it is unclear whether the introduction of rare earth elements into monolayer 2D-TMDCs can effectively control their carrier type and regulate carrier concentration.In this work, we demonstrate a large-sized MoS_2_ film doped with the RE element Sm by an atmospheric pressure, three-zone CVD method. In addition, stable monolayer triangular Sm-doped MoS_2_ films were obtained at the edge positions of the large size films.

MoS_2_ was chosen as the doping host material because it is a typical example from the layered 2D-TMDC family of materials [23]. Additionally, Sm is more economic compared to Er and Eu elements when investing in the optimization conditions for rare earth element-doped monolayer 2D materials by CVD. The monolayer MoS_2_ was used as a matrix material to embed Sm, as confirmed by characterization methods such as Raman, photoluminescence (PL), X-ray photoelectron spectroscopy (XPS), atomic force microscope (AFM), and energy dispersive X-ray spectroscopy (EDS) elemental mapping. As the monolayer triangle MoS_2_ was found to be the most energetically stable existent morphology, we characterized the electrical properties of stable triangular MoS_2_ field effect transistor (FET) before and after its doping. Electrical measurements showed that Sm element doping led to considerable changes in the electronic band structure of the host MoS_2_.The doping of Sm may lead to a non-uniform charge distribution, suppress the n-type characteristic, and change the energy band structure of MoS_2_.

## 2. Materials and Methods

### 2.1. Synthesis of the Sm-Doped MoS_2_ Film on a SiO_2_/Si Substrate by the CVD System

The three-zone CVD system is composed of two parts—an external temperature zone heated by a heating belt and the two temperature zones of a furnace. Alcohol/isopropanol/ deionized water and a 3:1 mix solution of concentrated H_2_SO_4_ and H_2_O_2_ were used in this study for the pretreatment of the 270 nm-thick, SiO_2_-capped Si substrate before Sm-doped MoS_2_ growth by CVD [24]. Sublimed sulfur powder (Aladdin, Shanghai, China, 99.5%; 900 mg), MoO_3_(VI) powder (Alfa, Louis, MO, USA, 99.5%; 10 mg), and SmCl_3_·6H_2_O particles (Macklin, Shanghai, China, 99%; 5 mg) were loaded in three customized crucible lids before growth and placed in the quartz tube of the furnace, as shown in the Supplementary Materials (Figure S1).

Then, the different samples in the three-crucible lid were fed by a tool into the corresponding positions of the quartz tube. First, the SiO_2_/Si (1 cm × 2 cm) substrate that was placed upside down on top of the MoO_3_ powder crucible lid was placed in the second zone of the furnace. Second, the SmCl_3_·6H_2_O particle was put in the first zone of the furnace. Third, sulfur powder was located at the external temperature zone. The temperature and Ar gas flow control procedure for CVD-doped growth is as follows. After purging the furnace with Ar for 20 min [25], the temperature of the two temperature zones of the tube furnace itself was ramped to 100 °C in 20 min and maintained for 30 min, while the carrier gas flow rate in this process was 200 sccm. The subsequent steps in the temperature and Ar gas flow control procedure were that the first and second temperature zones of the furnace were increased from 100 to 800 and 750 °C, respectively, within 53 min and then maintained for 30 min. The Ar gas flow rate changed from 200 to 100 sccm when heating up from 100 °C. Additionally, the S powder was heated by a heating belt when the first zone of the furnace was heated to 650 °C. After the thermal growth process ended, the furnace and heating belt were allowed to naturally cool down to room temperature [26].

### 2.2. FET Device Fabrication

The FET device preparation is divided into two main parts: sample transfer and source and drain electrode preparation [27,28]. During the transfer process, a thin layer of poly methyl methacrylate (PMMA) was spun-coated on the CVD-grown, Sm-doped MoS_2_ and then baked for 3 min at 150 °C. Next, the excess non-sample area was removed, and the remaining sample coated by the PMMA was immersed in 20% Hydrofluoric acid solution to etch SiO_2_. At the end, the detached film was cleaned in deionized water several times and transferred to the heavily doped SiO_2_/Si substrate. Next was the process of preparing the electrodes for the FET device. First, the residual PMMA covering the transfer sample on the target substrate was removed with an acetone solution. Second, a new thin layer of PMMA was spun-coated on the CVD-grown, Sm-doped MoS_2_, and then the electrodes were exposed in the appropriate positions via electron beam lithography (EBL) according to the designed electrode pattern. Third, Cr/Au (10 nm/50 nm) of the source and drain electrodes was deposited in the corresponding position by electron beam evaporation deposition. In SiO_2_/Si substrates, Cr/Au (10 nm/50 nm) electrode plating was also required for Si as a gate voltage. Finally, the acetone solution removed the PMMA, and the corresponding device was obtained on the substrate.

### 2.3. Characterization

Raman and PL spectra were taken by a LabRAM HR Evolution system (HORIBA Co. Ltd., Paris, France) with a 532 nm laser. The Raman spectroscopy parameters were a diffraction grating of 1800 gr/mm, a focal length of 800 mm, a Raman frequency shift range of 50–8000 cm^−1^, and a spectral resolution of ≤0.65 cm^−1^. The morphology of fabricated devices and the morphology of as-grown Sm-doped MoS_2_ were observed by a fluorescent inverted microscope (LeicaDMI6000B, Leica, Hesse-Darmstadt, Germany), and the thickness of few-layer flakes was characterized by AFM (MFP-3D-SA, Asylum Research, Santa Barbara, CA, USA) and Raman spectroscopy. The electrical properties of all the FET devices were measured using a Keithley 4200 (Tektronix, Beaverton, OR, USA) semiconductor parameter analyzer at room temperature (under dark conditions). XPS was conducted on a Thermo Scientific^TM^ K-Alpha^TM+^(Thermo Scientific, Waltham, MA, USA) spectrometer equipped with a monochromatic Al Kα X-ray source (1486.6 eV) operating at 100 W. Samples were analyzed under vacuum (*p* < 10^−8^ mbar) with a pass energy of 150 eV (survey scans) or 25 eV (high-resolution scans). All peaks would be calibrated with C1s peak binding energy at 284.8 eV for adventitious carbon. The experimental peaks were fitted with the Avantage software. TEM images were obtained with an FEI Talos F200X (Thermo Scientific, Waltham, MA, USA)

## 3. Results and Discussion

### 3.1. Fabrication of Monolayer Sm-Doped MoS_2_

**Figure 1:**
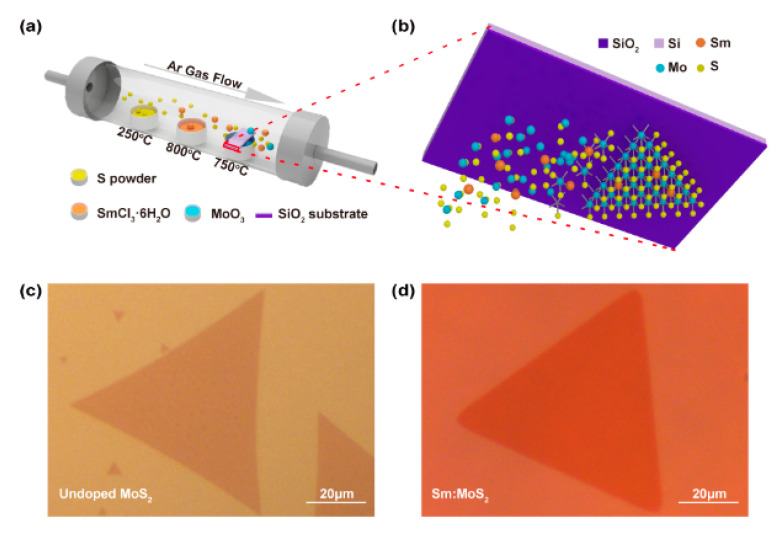
The growth of monolayer triangular Sm-doped MoS_2_ film. (**a**) Schematic of the three-temperature zone chemical vapor deposition (CVD) system for the growth of the monolayer Sm-doped MoS_2_ film on the SiO_2_/Si substrate. (**b**) The simple growth model processes for the synthesis of monolayer triangular Sm-doped MoS_2_ on SiO_2_/Si substrate. Optical microscopy images of (**c**) undoped and (**d**) Sm-doped monolayer triangular MoS_2_. Scale bar = 20 µm.

The monolayer Sm-doped MoS_2_ film was directly synthesized by the three-zone CVD system of atmospheric pressure method on the SiO_2_/Si substrate that was placed face down [29] on the crucible lid in a quartz tube, and the overall process of the growth system is illustrated in Figure 1a. As Figure 1a shows, sulfur powder was placed upstream, and the SmCl_3_·6H_2_O particle was placed in the middle of the S and MoO_3_ powder. In the synthetic process, SmCl_3_ has two effects: one is as a dopant, and the other is as an assistant agent for the long-distance transmission of MoO_3−x_ [24]. Additionally, the SiO_2_/Si (1 × 2 cm) substrate that was placed upside down on top of the MoO_3_ powder crucible lid was downstream of the furnace. Figure 1b shows an enlarged growth model processes for the synthesis of monolayer Sm-doped MoS_2_ on the SiO_2_/Si substrate facing down on the MoO_3_ powder. Figure 1c,d exhibits the optical images of the monolayer triangular MoS_2_ and Sm-doped MoS_2_, respectively. The size of CVD-grown, Sm-doped MoS_2_ triangle islands along the in-plane was up to around 50 µm. More optical images can be seen in Figure S2. The triangle MoS_2_ was the most energetically stable existent morphology, and the uniformity of the obtained large-size films was not as good as that of the triangle MoS_2_. The specific analysis can be seen in the Supplementary Materials (Figure S3).

### 3.2. Characterizations and Analysis of Monolayer Sm-Doped MoS_2_

#### 3.2.1. Raman and Photoluminescence Analysis of Monolayer Sm-Doped MoS_2_

**Figure 2:**
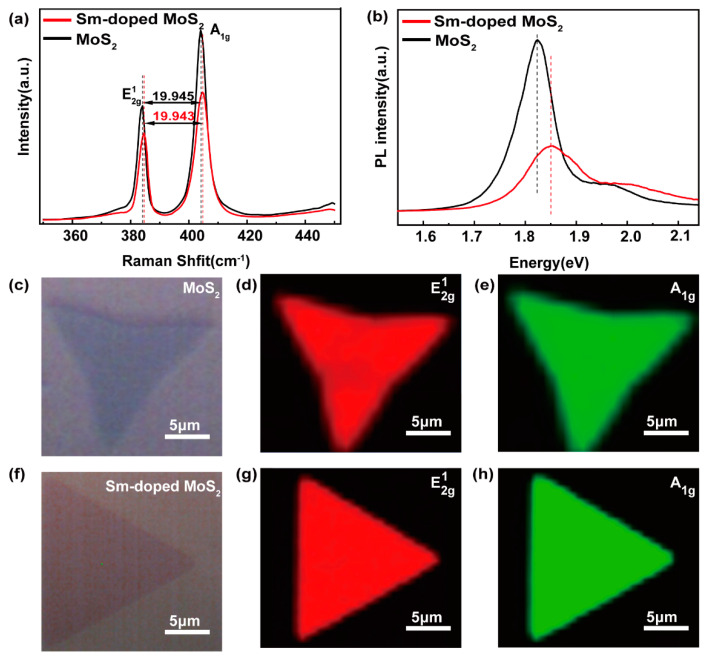
(**a**) Raman spectra and (**b**) photoluminescence (PL) spectra of monolayer triangular MoS_2_ and Sm-doped triangular MoS_2_ under 532 nm laser excitation. The (**c**) and (**f**) plots show the MoS_2_ and Sm-doped MoS_2_ optical microscopy images under Raman detection, respectively. (**d**,**e**,**g**,**h**) Raman mappings of the peak position corresponding to $E_{2g}^{1}$ and $A_{1g}$ of the MoS_2_ and Sm-doped MoS_2_, respectively. Raman and PL spectroscopy were conducted using a confocal Raman microscope with a 532 nm laser at room temperature.

Raman spectroscopy was performed to investigate the layers of the MoS_2_ and Sm-doped MoS_2_, and PL spectroscopy was employed to demonstrate the doping effect. Figure 2a shows the Raman spectra of MoS_2_ and Sm-doped MoS_2_, where the Raman spectrum of Sm-doped MoS_2_ can be seen to have exhibited two typical characteristic vibration modes of layered MoS_2_ at $E_{2g}^{1}$= 384.6 cm^−1^ (in-plane vibration of Mo and S atoms) and $A_{1g}$= 404.5 cm^−1^ (out-of-plane vibration of S atoms). The peak spacing between the $E_{2g}^{1}$ and $A_{1g}$ was 19.9 cm^−1^, suggesting the MoS_2_ and Sm-doped MoS_2_ were monolayers [30]. Figure S4b,d shows an AFM image of the Sm-doped MoS_2_ and undoped MoS_2_ samples, respectively. The height profiles indicate that the thickness of a typical flake was approximately 0.83 nm, which was almost comparable to that of the single layer MoS_2_ crystal. Additionally, both vibrational modes of Sm-doped MoS_2_ are displayed in a small blue shift compared with the monolayer MoS_2_, implying the possible effects of the Sm doping and/or the presence of defects on MoS_2_ [16]. Due to the competing effects of lattice strain and Sm^3+^ charge doping on the Raman shift, the overall blue shift of the $A_{1g}$ and $E_{2g}^{1}$ peak was relatively small [31]. Furthermore, the distortion of the MoS_2_ lattice could give rise to the optical quenching of the PL intensities [10]. It can be seen from Figure 2b that undoped MoS_2_ had a strong PL peak at about 1.82 eV, implying the MoS_2_ is a direct band gap semiconductor. However, the PL intensities of Sm-doped MoS_2_ were observed to be much lower with a 30 meV blue shift than that of undoped MoS_2_, which was consistent with previous predictions of the optical quenching of the PL intensities due to distortion of the MoS_2_ lattice [10]. This may be ascribed to the introduction of new defects due to lattice distortion by the doped Sm ions, as well as changes in band gap width. Using the equation $\delta \omega =2\gamma \omega_{0}\varepsilon$ [32] and band gap deformation potential, it was found that the lattice strains arising from the Sm doping were about 0.076% and 0.1%, respectively. This was also confirmed in the previous studies of lanthanide Er-doped [16] MoS_2_ and Eu-doped [22] MoS_2_. Additionally, the Raman mapping corresponding to the $E_{2g}^{1}$ and $A_{1g}$ peaks was used to confirm the uniformity growth of the monolayer triangular samples with and without Sm doping, as shown in Figure 2d,e,g,h, respectively. It was observed that the Sm-doped monolayer MoS_2_ showed a more regular triangular shape and a more uniform peak distribution than undoped MoS_2_. Thus, Sm doping improved the homogeneity of the sample.

#### 3.2.2. XPS Spectrum Analysis of Monolayer Sm-Doped MoS_2_

**Figure 3:**
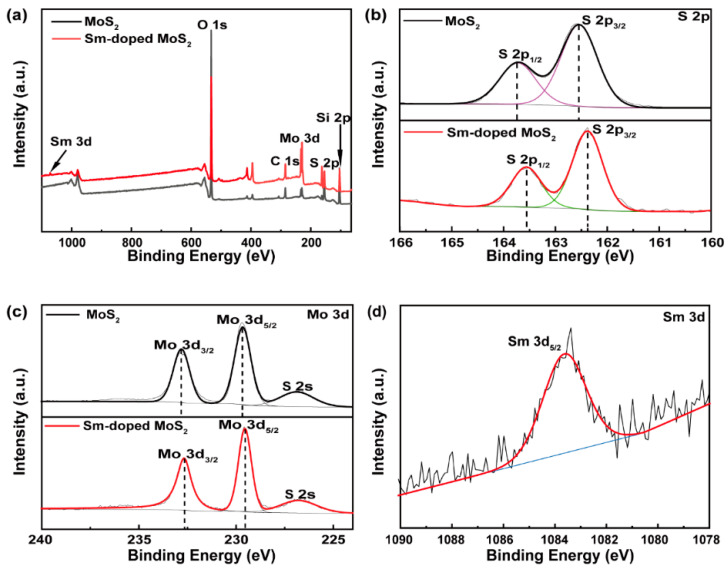
XPS spectrum (**a**) total scans, (**b**) S 2p, (**c**) Mo 3d, and (**d**) Sm 3d core levels.

XPS was performed to investigate the elements and valence state information of monolayer MoS_2_ and Sm-doped MoS_2_. Figure 3a–c displays the comparisons of XPS results from the undoped MoS_2_ (in black) and Sm-doped MoS_2_ (in red). In the full spectrum (Figure 3a), there are Si, O, and C core levels in addition to S and Mo core levels. The Si and O core levels in the samples may have come from the SiO_2_/Si substrate, so the O peak intensity was much higher than that of C. The core level of Sm was only found in the Sm-doped MoS_2_. Additionally, the spectral shape of the S and Mo core levels after Sm-doping was nearly identical to that of the undoped MoS_2_, which indicated that Sm-doping did not significantly chemically alter the MoS_2_ host [33]. However, compared to the undoped sample, it was obvious that the doped S 2p and Mo 3d core level peaks were both shifted by 0.18 eV towards the lower binding energies. The specific displacement changes of the binding energy were as follows: Mo 3d_3/2_ shifted from 232.64 to 232.82 eV, Mo 3d_5/2_ shifted from 229.7 to 229.52 eV, S 2p_1/2_ shifted from 163.74 to 163.56 eV, and S 2p_3/2_ shifted from 162.54 to 162.36 eV [34]. The slight shift indicated the change of chemical microenvironment around these atoms for Sm-doped MoS_2_ materials [22]. It can be inferred that the binding energy shifts were associated with Fermi level shifts and originated from the incorporation of Sm into MoS_2_ single crystals [16,22]. The binding energy shift direction and magnitude of Sm-doped MoS_2_ were also found to be consistent with the variation of p-type-doped MoS_2_ [16]. As seen in Figure 3d, the obvious binding energy peaks related to the Sm 3d_5/2_ at 1083.58 eV [35] indicated that the Sm ions were trivalent [36], but the Sm 3d_3/2_ did not have a significant peak. That may be attributed to the low concentration of Sm, with XPS estimating a doping concentration of about 1.1at%.

#### 3.2.3. TEM Analysis of Monolayer Sm-Doped MoS_2_

**Figure 4:**
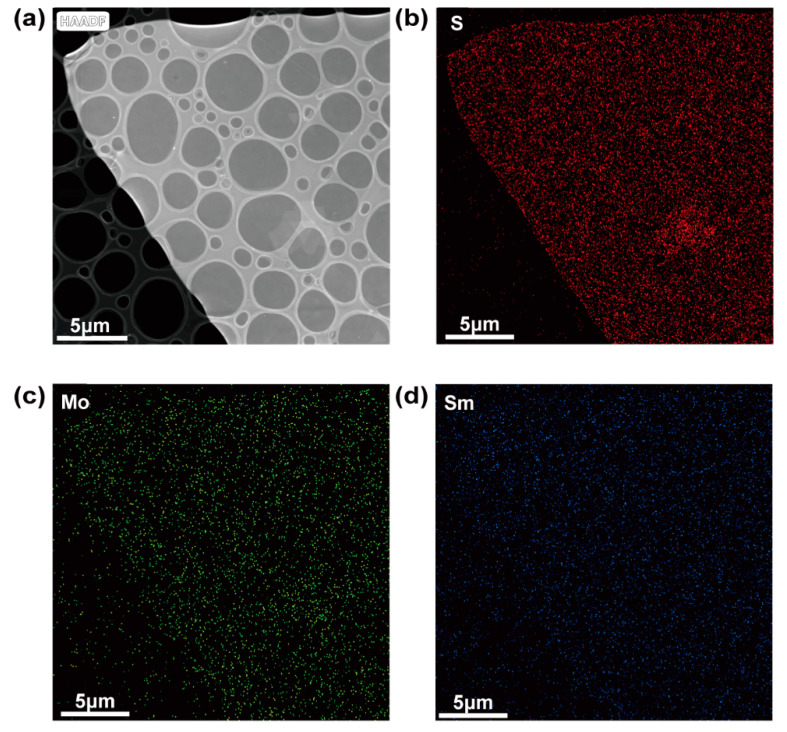
TEM characterizations of Sm-doped MoS_2_. (**a**) Low resolution TEM area of EDS mapping. (**b**–**d**) EDS elemental mapping images of S, Mo, and Sm in Sm-doped MoS_2_, respectively.

TEM was carried out to further investigate the microstructure and elemental composition of the monolayer Sm-doped MoS_2_. The EDS elemental mapping images of S, Mo, and Sm in Sm-doped monolayer MoS_2_, which were taken from the area shown in Figure 4a, are shown in Figure 4b–d. Though the S, Mo, and Sm elemental mapping image scans were complete, the amount of detected Sm element was small. This indicated the existence of a low doping concentration of Sm in the monolayer MoS_2_. The elemental Sm doping concentration of the selected area detected by EDS (Figure S5) was about 1.42at%. In Figure S5, selected area electron diffraction (SAED) patterns indicate that Sm doping did not change the original crystal structure.

### 3.3. Electrical Properties Characterisation of Monolayer Sm-Doped MoS_2_ FET

**Figure 5:**
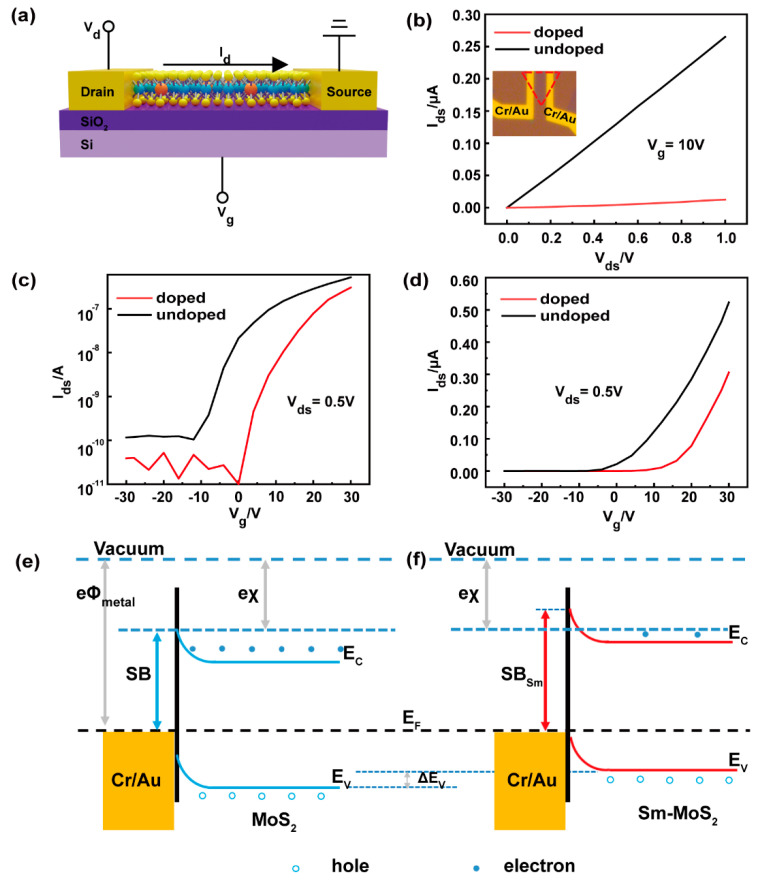
Electrical properties of monolayer MoS_2_ and Sm-doped MoS_2_. (**a**) The schematic drawing of a Sm-doped monolayer MoS_2_ FET. (**b**) Output curves (*I_ds_*–*V_ds_*) of MoS_2_ and Sm-doped MoS_2_ devices at a gate bias of 10 V. The inset is the topography of the device. (**c**) Source-drain current (*I_ds_*) vs gate voltage (*V_g_*) characteristics of monolayer MoS_2_ and the Sm-doped MoS_2_ FET device at a drain voltage (*V_ds_*) of 0.5 V on a log scale. (**d**) Transfer curves (*I_ds_*–*V_g_*) of monolayer MoS_2_ and the Sm-doped MoS_2_ FET device with a *V_g_* from −30 to 30 V. Band alignment diagrams and the formation of the Schottky barrier after contact between the Cr/Au electrode and (**e**) pristine MoS_2_ (blue line) and (**f**) Sm-doped MoS_2_ (red line).

In order to investigate the electrical properties of the Sm-doped monolayer MoS_2_, a bottom gate FET was fabricated on the SiO_2_/Si substrate with Cr/Au (10/50 nm) as the source and drain electrodes as shown in Figure 5a. The SiO_2_ layer thickness of the FET devices was 285 ± 20 nm. Specifically, the electrical performance of the FET devices was tested in the dark. Figure 5b shows the output curves of Sm-doped (red) and undoped (black) monolayer MoS_2_ at a gate voltage of 10 V. The inset shows a microscopy image of the FET under the light microscope. It is clear that the output characteristics of monolayer MoS_2_ were better than those of Sm doping under the same conditions, implying the restraint of electrical conductivity with Sm doping. This was due to the fact that the Sm element caused distortions in the lattice structure near the doping site, resulting in an inhomogeneous charge density distribution. Figure 5c,d shows the transfer curves of an MoS_2_ FET with and without Sm doping. The undoped MoS_2_ FET displayed typical n-type transport characteristics (black), as previously reported in the literature [23,37,38,39]. Compared to undoped MoS_2_ (red), the threshold voltage (*V*_th_) of Sm-doped MoS_2_ moved from −12 to 0 V, as seen in Figure 5c. It can also be seen in Figure 5d that the inflection point of the transfer curve of the doped MoS_2_ shifted to a more positive gate voltage with respect to the inflection point of the undoped MoS_2_, implying a Fermi level closer to the valence band maximum (VBM) by Sm doping. More *V*_ds_ transfer characteristics of Sm-doped MoS_2_ FET devices are shown in Figure S6 in the Supplementary Materials, where it can be seen that the *V*_th_ for doping all showed a tendency to shift towards a more positive gate voltage. The positive shift of the *V*_th_ was consistent with the p-type dopant behavior of in MoS_2_ [40]. This was because during the Sm substitution doping process, the Sm 4*f* states contributed to the formation of a valence band and a hybridization of Sm 4*f* and Mo 4*d* on the edge of the valence band. This brought the Fermi energy level close to the valence band, leading to p-type doping. Furthermore, this was also coherent with the p-type doping conclusion obtained from the core energy level of XPS that shifted towards a lower binding energy. However, significant p-type (hole-transport) behavior of MoS_2_ devices was not observed, presumably due to the Fermi-level pinning of the Cr/Au contact metal close to the conduction band of MoS_2_ [33,41] or a smaller shift in the Fermi energy level caused by a smaller doping concentration. Figure 5e, f shows the band alignment diagrams and the formation of the Schottky barrier (SB) before and after doping. The figure shows the schematic of an energy diagram of a Cr/Au electrode and an MoS_2_ monolayer where the work functions of Cr (Φ_Cr_) and Au (Φ_Au_) were 4.5 and 5.1 eV, respectively [38], and the electron affinity (χ) of MoS_2_ was ∼4.2 eV [42]. The energy band of the MoS_2_ bended upon contact to form the SB (blue line). Sm doping in MoS_2_ caused the Fermi energy to shift closer to the VBM and the impurity level to be close to the VBM. Additionally, an empty Sm 4*f* state was present at the bottom of the conduction band. All of this led to the Fermi energy level being closer to valence band and an increase the height of the SB_Sm_, as well as a decrease in device current. Moreover, this corresponded to the actual measured electrical properties. The field-effect mobility (*μ_FE_*) could be calculated from transfer curve (Figure 5d) according to the following equation [39]:

(1) $$\mu_{FE}=\frac{{dI}_{ds}}{{dV}_{g}}\cdot \frac{L}{W}\cdot \frac{1}{C_{g}{\cdot V}_{d}}$$

(2) $$C_{g}=\frac{\varepsilon_{0}\varepsilon_{r}}{d}$$

where *dI_ds_*/*dV_g_* is the slope of the transfer curve; *L* and *W* are the length and width of the channel, respectively; *V_d_* is the drain voltage; *C_g_* is area-normalized capacitance of 280 nm-thick SiO_2_; $\varepsilon_{0}$ is vacuum dielectric constant; $\varepsilon_{r}\;$is relative dielectric constant; and *d* is the SiO_2_ thickness. The calculation gave mobility values of 4.35 and 3.08 cm^2^/Vs for Sm-doped and undoped uncased monolayer FETs, respectively. Furthermore, the on/off current ratio for *V_g_* ranged from −30 to +30 V, with a source-drain bias of 0.5 V for the Sm-doped monolayer MoS_2_ FET of about $3\times 10^{4}$, which was higher than that of the undoped monolayer MoS_2_—$5\times 10^{3}$. This represented a 500% improvement in the on/off performance of the doped device, but the change in migration rate was not very significant. The *I_ds_*–*V_g_* curve in Figure 5c indicates that the FET made of Sm-doped MoS_2_ showed a current density close to the MoS_2_ FET at the “on” state but a much lower current density (1000 times lower) at the “off” state. These results suggested that Sm doping reduced the electron density in MoS_2_ and led to a small *I_off_*. As a result, both the radius difference and concentration of impurities can affect the electrical properties of monolayer MoS2. This is because the lattice distortion caused by the doped atoms can change the energy band structure of monolayer MoS_2_ and affect the charge distribution, and the doping concentration determines the variation in band gap width and Femi energy level.

## 4. Conclusions

In conclusion, Sm-doped monolayer triangular MoS_2_ was successfully grown by CVD, which was confirmed to be monolayer by Raman and AFM measurements. FET devices were fabricated with the Sm-doped monolayer triangular MoS_2_, and its *V*_th_ was shifted from −12 to 0 V compared to be undoped, MoS_2_-based FETs. The positive shift change in *V*_th_ was attributed to the fact that Sm acted as substitutional p-type dopant in MoS_2_, consistent with theoretical predictions and XPS analysis. The 500% increase in the on/off current ratio of Sm-doped devices was due to the reduction of the electron density in MoS_2_ after Sm ion doping, which also led to a small *I_off_*. This was due to the lattice strain caused by Sm doping. The above results show that RE element Sm substitutional doping can tune and enhance the electrical properties of monolayer MoS_2_. This study opens up a wide range of applications for tuning the electrical properties of monolayer 2D-TMDCs by doping with RE elements.

## Supplementary Materials

The following are available online at https://www.mdpi.com/2079-4991/11/3/769/s1. Figure S1: Schematic of the CVD system for growth of monolayer Sm-doped MoS_2_ film on SiO_2_/Si substrate. Figure S2: Optical image of triangular monolayer Sm-doped MoS_2_. Figure S3: Disordered and irregular films during CVD doping growth and the respective corresponding Raman spectra. Figure S4: Optical microscopy images of (a) Sm-doped and (c) undoped MoS_2_ triangles. AFM images and the height of (b) Sm-doped and (d) undoped MoS_2_. Figure S5. (a) Low-magnification TEM image of Sm-doped MoS_2_ single crystal on a Cu grid. SAED patterns (b,c) collected from different sites on the monolayer triangle Sm-doped MoS_2_. (d) Low-magnification TEM image of Sm-doped MoS_2_; the red box shows the measured area of figure (e). EDS spectrum (e) of Sm-doped MoS_2_ with Mo, Sm, and S labels and inset showing the atomic percent of labeled elements. Inset: partial enlargement. Figure S6: (a) Photomicrograph of the FET device. (b) Transfer curves (*I_ds_*–*V_g_*) of monolayer MoS_2_ FET device at drain voltages (*V_ds_*) of 0.1, 1, and 2 V with *V_g_* varying from −30 to 30 V. (c) Transfer curves (*I_ds_*–*V_g_*) of monolayer Sm-doped MoS_2_ FET device at drain voltages (*V_ds_*) of 0. 1, 1, and 2 V with *V_g_* varying from −30 to 30 V. Transfer curves (*I_ds_*–*V_g_*) comparing of monolayer MoS_2_ and Sm-doped MoS_2_ at drain voltages (*V_ds_*) 2 V (d), 1 V (e), and 0. 1 V (f) with *V_g_* varying from −30 to 30 V.
