# Supplementary material for: Enhanced Electrical Performance of Monolayer MoS2 with Rare Earth Element Sm Doping
Source: Nanomaterials (Basel). 2021 Mar 18;11(3):769. doi: 10.3390/nano11030769 (PMC8002856; doi:10.3390/nano11030769)
Supplement: Supplementary file 1 [file nanomaterials-11-00769-s001.pdf]

Supplementary Materials

Enhanced Electrical Performance of Monolayer MoS_2 with Rare Earth Element Sm Doping

Shijie Li [1, 2, †], Shidai Tian [1, 2, †], Yuan Yao[1, 2], Meng He[1, 2], Li Chen[1, *], Yan Zhang[1, 2, 3, *], Junyi Zhai [1, 2, 4, *]

[1] Center on Nanoenergy Research, School of Physical Science and Technology, Guangxi University, Nanning, 530004, China; lishijie@binn.cas.cn (S.L.); tianshidai@binn.cas.cn (S.T.) yaoyuan@bin-n.cas.cn (Y.Y.); hemeng@binn.cas.cn (M.H.)

[2] CAS Center for Excellence in Nanoscience, Beijing Key Laboratory of Micro-nano Energy and Sensor, Beijing Institute of Nanoenergy and Nanosystems, Chinese Academy of Sciences, Beijing, 100083, China;

[3] School of Physics, University of Electronic Science and Technology of China, Chengdu, 610054, China

[4] College of Nanoscience and Technology, University of Chinese Academy of Science, Beijing, 100049, China

* Correspondence: chenli@gxu.edu.cn (L.C.); zhangyan@uestc.edu.cn (Y.Z.); jyzhai@binn.cas.cn (J.Z.)

† These authors contributed equally to this work.

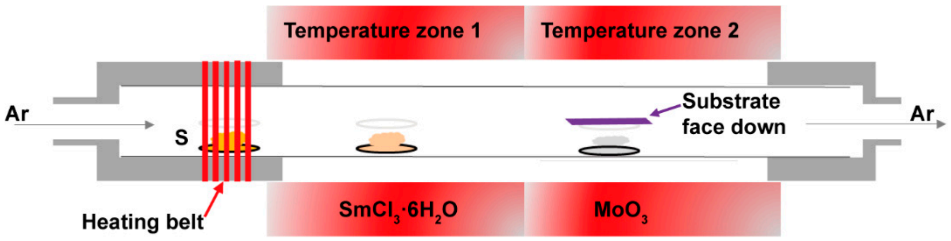

Figure S1. Schematic of the CVD system for growth of monolayer Sm-doped MoS_2 film on SiO_2/Si substrate.

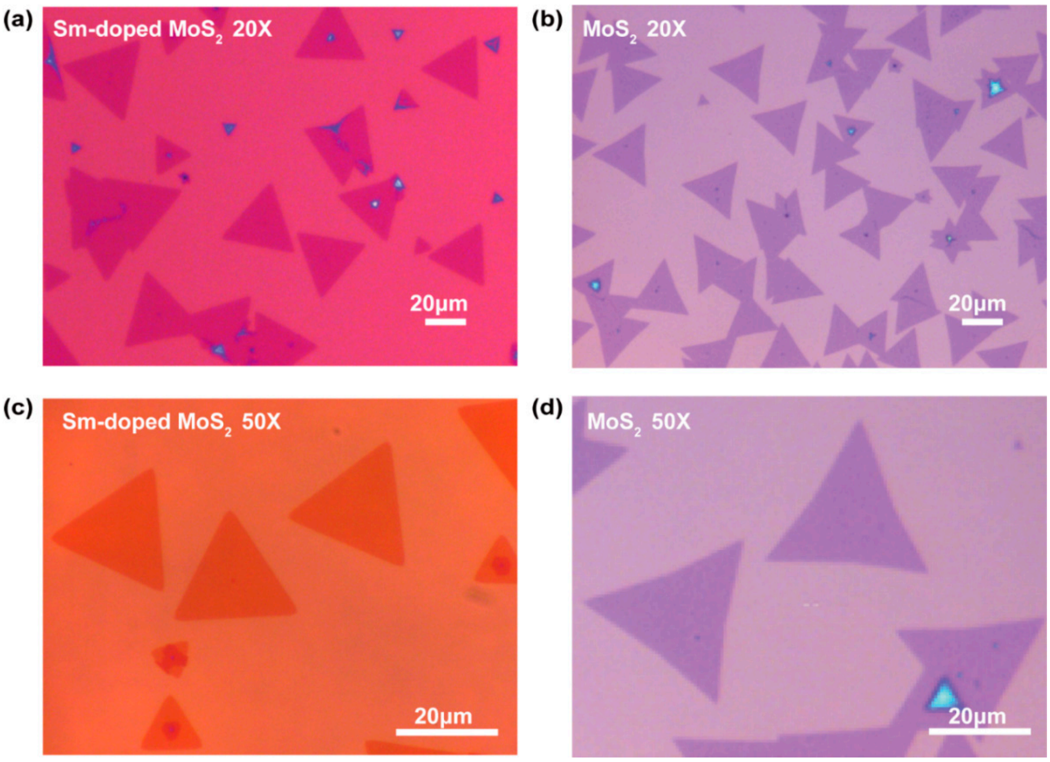

Figure S2. Optical image of triangular monolayer Sm-doped MoS_2 (a) and triangular monolayer MoS_2(b) islands CVD-grown on SiO_2/Si substrates on a Si substrate under a 20X optical microscope. Optical image of triangular monolayer Sm-doped MoS_2 (c)and triangular monolayer MoS_2(d) islands CVD-grown on SiO_2/Si substrates on a Si substrate under a 50X optical microscope.

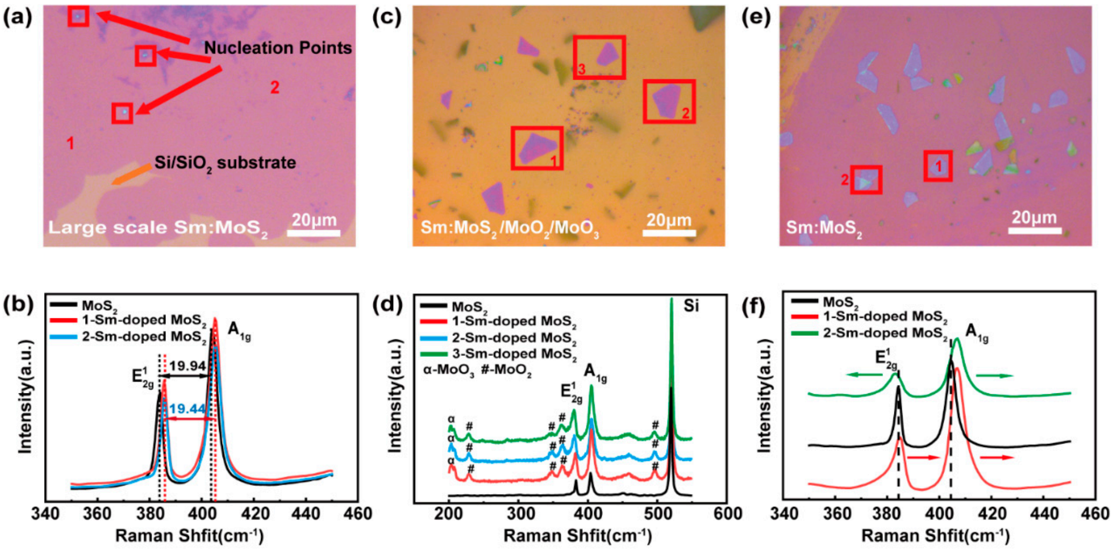

Figure S3. Disordered and irregular films during CVD doping growth, and the respective corresponding Raman spectra. (a) Optical image of CVD grown a large scale continuous Sm-doped MoS_2 film on SiO_2/Si substrates. (b) Raman spectra comparing of triangular monolayer MoS_2 and large scale Sm-doped MoS_2. (c) Optical image of CVD grown irregular Sm-doped MoS_2 or MoO_2 nanosheets on SiO_2/Si substrates. (d) Raman spectra comparing of triangular monolayer MoS_2 and large irregular Sm-doped MoS_2 or MoO_2 nanosheets. (e) Optical image of CVD grown irregular Sm-doped MoS_2 film on SiO_2/Si substrates. (f) Raman spectra comparing of triangular monolayer MoS_2 and large irregular Sm-doped MoS_2 film.

As shown in the Figure S3, a number of additional doped materials such as large-size film (a), thicker precursor nanosheets (c) and polygonal thin films were obtained (e) by CVD growth. Multiple nucleation sites can be found in the continuous films in Figure S3 (a). And two sites were randomly selected for Raman's characterization in the large-size Sm-doped MoS_2 films. A small blue shift in large-size Sm-doped MoS_2 is consistent with the change in monolayer triangular Sm-doped MoS_2. However, this continuous film was made up of a larger number of nucleation sites joined together by covering each other during growth, so the thickness is not very uniform. The irregular nanosheets in S3 (c) show the intermediate products grown by Raman peaks for in situ doping: Sm: MoO_2 [1] and MoO_3 [2]. These results show a variation and instable of vapor pressure along the flow direction with CVD system of atmospheric pressure method. Yet, it does not affect the acquisition of monolayers triangle of stable Sm-doped MoS_2. Figure S3 (e) shows a Sm-doped MoS_2 multilayer irregular film. Two characteristic peaks were measured (f) at position 2, E_{2g}^{1} peak shifted to the left and A_{1g} peak shifted to the right, which is consistent with the variation in the displacement of the multilayer MoS_2 characteristic peak.

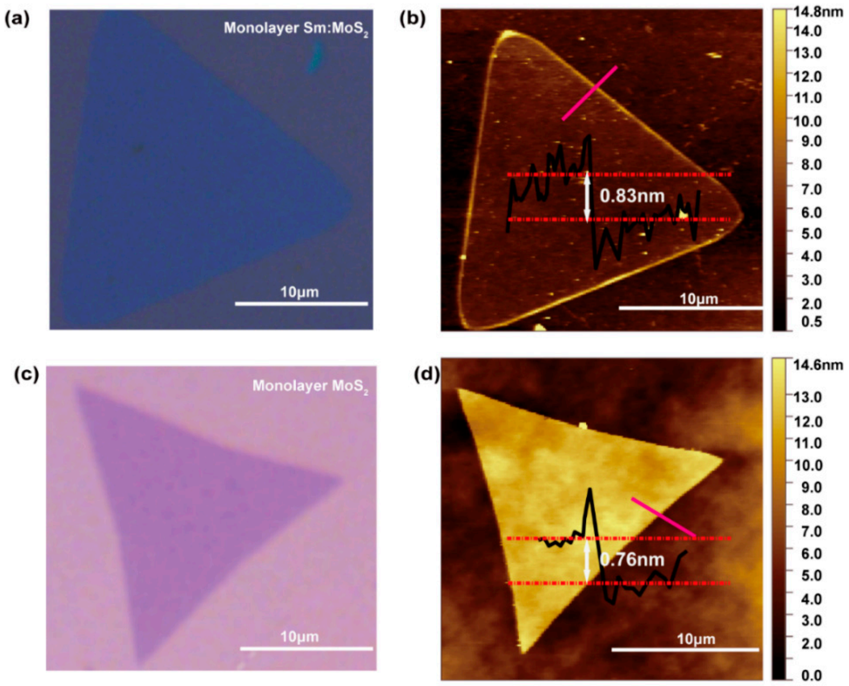

Figure S4. Optical microscopy images of (a) Sm-doped and (c) undoped MoS_2 triangles. AFM images and the height of (b) Sm-doped and (d) undoped MoS_2. Scale bars: 10µm.

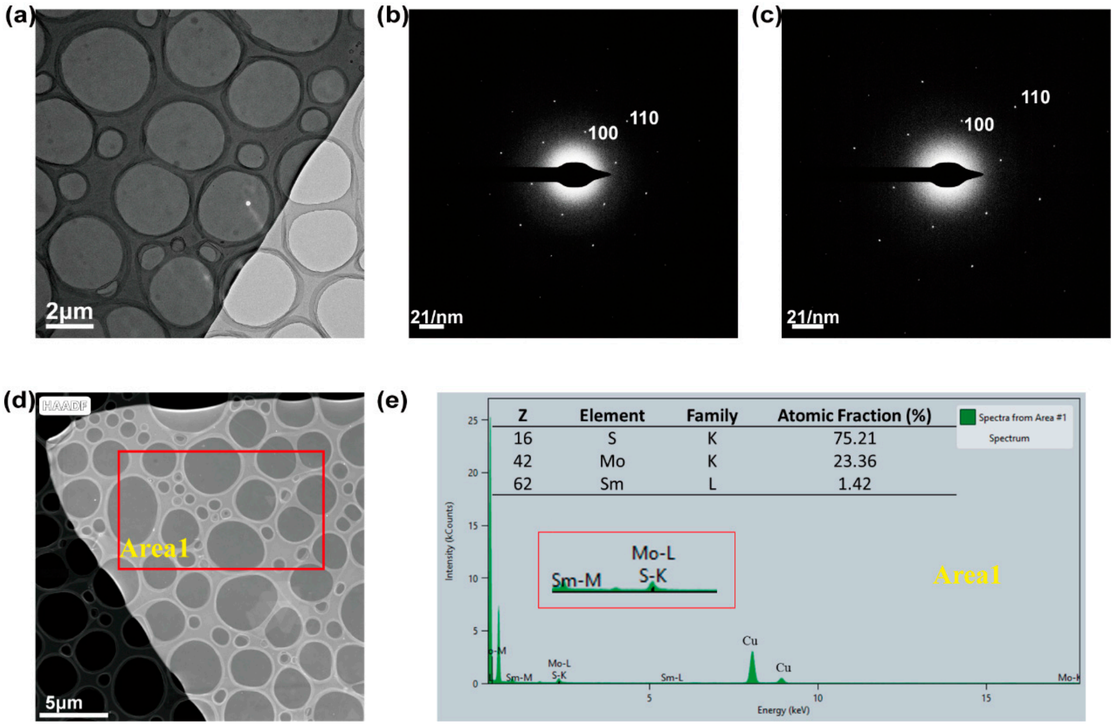

Figure S5. (a) Low-magnification TEM image of Sm-doped MoS_2 single crystal on a Cu grid. SAED patterns (b-c) collected from different sites on the monolayer triangle Sm-doped MoS_2. (d) Low-magnification TEM image of Sm-doped MoS_2, the red box shows the measured area of figure (e). EDS spectrum (e) of Sm-doped MoS_2 with Mo, Sm, and S labeled and inset showing the atomic percent of

labeled elements. Inset: Partial enlargement.

The selected area electron diffraction (SAED) patterns indicate that Sm doping does not change the original crystal structure. The elemental Sm doping concentration of selected area detected by EDS spectrum was about 1.42at%.

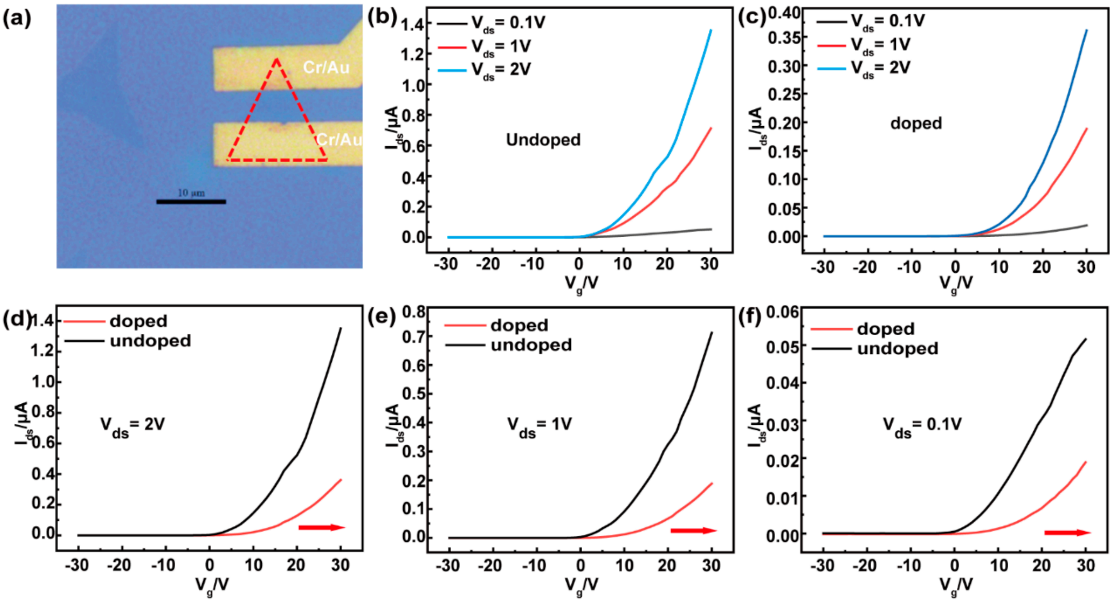

Figure S6. (a)Photomicrograph of the FET device. (b)Transfer curves (I_{ds}-V_g) of monolayer MoS_2 FET device at drain voltages (V_{ds}) 0.1V, 1V, 2V with V_g varying from -30 V to 30 V. (c) Transfer curves (I_{ds}-V_g) of monolayer Sm-doped MoS_2 FET device at drain voltages (V_{ds}) 0. 1V, 1V, 2V with V_g varying from -30 V to 30 V. Transfer curves (I_{ds}-V_g) comparing of monolayer MoS_2 and Sm-doped MoS_2 at drain voltages (V_{ds}) 2V (d), 1V(e), 0. 1V(f) with V_g varying from -30 V to 30 V.

References

1. Özden, A.; Ay, F.; Sevik, C.; Perkgöz, N.K., CVD growth of monolayer MoS_2: Role of growth zone configuration and precursors ratio. *Japanese Journal of Applied Physics* **2017,** *56* (6S1).

2. Liu, H.F.; Wong, S.L.; Chi, D.Z., CVD Growth of MoS_2-based Two-dimensional Materials. *Chemical Vapor Deposition* **2015,** *21* , 241–259.
